# Body mass index and chronic kidney disease outcomes after acute kidney injury: a prospective matched cohort study

**DOI:** 10.1186/s12882-021-02400-3

**Published:** 2021-05-28

**Authors:** Helen L. MacLaughlin, Mindy Pike, Nicholas M. Selby, Edward Siew, Vernon M. Chinchilli, Andrew Guide, Thomas G. Stewart, Jonathan Himmelfarb, Alan S. Go, Chirag R. Parikh, Nasrollah Ghahramani, James Kaufman, T. Alp Ikizler, Cassianne Robinson-Cohen, for the ASSESS-AKI Study Investigators, Vernon M. Chinchilli, Alan S. Go, Jonathan Himmelfarb, T. Alp Ikizler, James S. Kaufman, Paul L. Kimmel, Chirag R. Parikh, John B. Stokes, Steven Coca, Amit Garg, Chi-yuan Hsu, Raymond K. Hsu, Kathleen D. Liu, Nasrollah Ghahramani, W. Brian Reeves, Edward D. Siew, Julia B. Lewis, Lorraine Ware, Prasad Devarajan, Catherine Krawczeski, Michael Bennett, Michael Zappitelli, Mark Wurfel

**Affiliations:** 1 Queensland University of Technology, School of Exercise and Nutrition Sciences, Victoria Park Road, Kelvin Grove, QLD Australia; 2 Royal Brisbane and Women’s Hospital, Herston, QLD Australia; 3 Division of Nephrology, Vanderbilt University Medical Center, Nashville, TN USA; 4 Centre for Kidney Research and Innovation, University of Nottingham, Derby, UK; 5 Division of Biostatistics and Informatics, Pennsylvania State University, Hershey, PA USA; 6 Department of Biostatistics, Vanderbilt University School of Medicine, Nashville, TN USA; 7 Division of Nephrology, University of Washington, Seattle, WA USA; 8 Kaiser Permanente Northern California, Oakland, CA USA; 9 University of California, San Francisco, San Francisco, CA USA; 10 Division of Nephrology, Johns Hopkins School of Medicine, Baltimore, MD USA; 11 Division of Nephrology, Department of Medicine, Penn State College of Medicine, Hershey, PA USA; 12 Renal Section, Veterans Affairs New York Harbor Health Care System and New York University School of Medicine, New York, NY USA

**Keywords:** Kidney, Body mass index (BMI), Mortality, Obesity

## Abstract

**Background:**

Acute kidney injury (AKI) and obesity are independent risk factors for chronic kidney disease (CKD). This study aimed to determine if obesity modifies risk for CKD outcomes after AKI.

**Methods:**

This prospective multisite cohort study followed adult survivors after hospitalization, with or without AKI. The primary outcome was a combined CKD event of incident CKD, progression of CKD and kidney failure, examined using time-to-event Cox proportional hazards models, adjusted for diabetes status, age, pre-existing CKD, cardiovascular disease status and intensive care unit admission, and stratified by study center. Body mass index (BMI) was added as an interaction term to examine effect modification by body size.

**Results:**

The cohort included 769 participants with AKI and 769 matched controls. After median follow-up of 4.3 years, among AKI survivors, the rate of the combined CKD outcome was 84.7 per1000-person-years with BMI ≥30 kg/m^2^, 56.4 per 1000-person-years with BMI 25–29.9 kg/m^2^, and 72.6 per 1000-person-years with BMI 20–24.9 kg/m^2^. AKI was associated with a higher risk of combined CKD outcomes; adjusted-HR 2.43 (95%CI 1.87–3.16), with no evidence that this was modified by BMI (*p* for interaction = 0.3). After adjustment for competing risk of death, AKI remained associated with a higher risk of the combined CKD outcome (subdistribution-HR 2.27, 95%CI 1.76–2.92) and similarly, there was no detectable effect of BMI modifying this risk.

**Conclusions:**

In this post-hospitalization cohort, we found no evidence for obesity modifying the association between AKI and development or progression of CKD.

**Supplementary Information:**

The online version contains supplementary material available at 10.1186/s12882-021-02400-3.

## Introduction

An increasing prevalence of diabetes, proteinuria, chronic kidney disease (CKD) and obesity, together with an aging population at higher risk for hospitalizations, are likely contributors to the increasing incidence of acute kidney injury (AKI) [1]. Both experimental animal studies and human observational cohorts suggest an increased risk of permanent kidney damage, manifested as CKD, after an episode of AKI [2–8]. However, the retrospective nature, and its attendant methodological limitations, of studies examining this question constrain our understanding of the external validity and determinants of this association. Obesity is a risk factor for the development and progression of CKD [9, 10], independent of the related risk factors hypertension and diabetes [11–13]. Obesity is also associated with both a higher risk, and greater severity of AKI [14–16], but may be protective for early post-AKI survival up to 90-days [17–19].

Mechanisms including renal hypoxia and inflammation may drive the transition from AKI to CKD [20, 21]. Obesity is also a chronic pro-inflammatory state, [22, 23] and studies demonstrate reductions in markers of inflammation with weight loss, both in CKD and non-CKD populations [23–26]. Proximal tubule hypoxia has been demonstrated in an obesity induced kidney injury mouse model [24]. These pre-existing adaptations associated with obesity, may potentially influence the repair/remodeling process in kidney tubules and exacerbate the risk for declining kidney function after AKI. To date, no studies have examined the effect of obesity, or body mass index, on the development or progression of CKD, end-stage kidney disease, or cardiovascular events, in a cohort followed after hospitalization, with, or without AKI.

The ASessement, Serial Evaluation and Subsequent Sequelae in Acute Kidney Injury (ASSESS-AKI) study is a multi-center prospective study that examined the risk of CKD development and progression and other clinical outcomes after an episode of hospitalized AKI. It is the only such prospective study including protocolized measurements of height and weight in a prospective cohort of hospitalized patients matched for baseline CKD status, with and without AKI. The purpose of this investigation is to evaluate whether obesity modifies the relationship between AKI and CKD outcomes in a matched cohort of participants hospitalized with and without AKI. We hypothesize that those with high BMI are a higher risk sub-set for subsequent CKD development or progression after an episode of AKI. Furthermore, we hypothesize that obesity is a risk factor for the development or progression of CKD in this hospital-based cohort, with, or without AKI.

## Methods

Study population: The ASSESS-AKI parallel matched cohort study includes hospitalized adult participants with or without an AKI who survived and completed a baseline study visit 3 m after hospital discharge. The full study methods, including detailed inclusion and exclusion criteria, and definitions of covariates, and the primary outcomes have been published previously [27]. The study was approved by the Institutional Review Boards of Vanderbilt University, Yale University, Pennsylvania State University, and Kaiser Permanente and was conducted in accordance with the Declaration of Helsinki; written informed consent was obtained from participants. Data were collected prospectively from participants enrolled from four North American clinical centers between December 2009 and February 2015 and up until the end of follow up in November 2018. Acute kidney injury was defined using the Kidney Disease Improving Global Outcomes (KDIGO) criteria of an increase in inpatient serum creatinine of at least 50% or ≥ 0.3 mg/dL above the most recent, non-emergency department creatinine obtained between 7 and 365 days previously [28]. Participants with AKI, were matched to a participant without AKI for study center, pre-AKI chronic kidney disease defined as estimated glomerular filtration rate (eGFR) of < 60 ml/min/1.73m^2^ calculated from serum creatinine using the CKD-EPI equation [29], age, pre-AKI diabetes status, previous cardiovascular disease (CVD), stage of pre-AKI kidney function, and admission to an intensive care unit (ICU) during the index hospitalization.

### Data collection

Time of entry into the study was the date of AKI diagnosis in the AKI group and the date of index hospital discharge in the non-AKI group. Full eligibility was confirmed, and height and weight were measured, at the baseline study visit, 3 months post discharge from the index hospitalization. Study visits were conducted annually from the index hospitalization until the end of follow up, death, or censoring due to loss to follow up or participant withdrawal. Data collected at annual visits included measurement of kidney function using the CKD-EPI equation with serum creatinine [29] to determine new or progressing CKD, interim hospital admissions, alterations to medication and updated medical history, including major events.

Height was measured, in centimeters, using a wall mounted stadiometer, or portable stadiometer with the baseplate placed against a wall. Participants stood against the stadiometer with the back, buttocks and heels touching the stand or backplate, arms and shoulders relaxed and the head in the Frankfurt plane so that the orbitale and the tragus were aligned horizontally, and the headplate was lowered to rest on the top of the head. Participants were weighed in their own clothes, with an empty bladder, and after removing items such as belts and jewellery. Weight was determined using a calibrated electronic scale (model UC-321PL; A&D Medical, CA). Weight was measured twice, with the first measure recorded if the second measure was within 0.1 kg of the first. If the difference between the two measures was more than 0.1 kg, weighing was repeated until 2 measures were within 0.1 kg and the first of these was recorded.

### Body mass index (BMI)

BMI was calculated as weight in kilograms divided by height in meters squared. BMI was examined continuously, per kg/m^2^, and categorically, with obesity defined as BMI ≥ 30 kg/m^2^, and overweight defined as 25–29.9 kg/m^2^ according to the World Health Organisation (WHO) classification. Normal weight was defined as BMI 20–24.9 kg/m^2^ which is within the WHO normal weight range [30] and this was considered the a priori referent category. BMI < 20 kg/m^2^ was classified as underweight.

### Outcomes

Study coordinators screened for major events (hospitalizations, procedures, dialysis) from medical records and participant self-report at study visits. Kidney disease outcomes were calculated from serum creatinine-based estimated GFR obtained at annual study visits. The primary outcome was a combined CKD event comprised of incident CKD, CKD progression or the development of end stage renal disease (ESRD). In those without CKD at enrollment, incident CKD was defined as experiencing both a minimum 25% reduction in level of eGFR compared with pre-enrollment baseline eGFR and achieving CKD Stage 3 or worse during follow-up [31]. CKD progression, in those with pre-existing CKD at index hospitalization, was defined as a greater than 50% reduction in level of eGFR compared with baseline, or progressing to an eGFR less than 15 ml/min/1.73m^2^. Development of end stage renal disease (ESRD) was defined as any of the following: (1) peritoneal dialysis or hemodialysis treatment at least once a week for at least 12 consecutive weeks, (2) receipt of a kidney transplant and/or (3) death while receiving dialysis.

Death was ascertained by review of medical records or death certificates if available, or through cross checking with the National Death Index by social security number for participants lost to follow up. Major atherosclerotic cardiovascular events (MACE) of myocardial infarction, ischemic stroke and peripheral artery disease were self-reported or recorded from electronic medical records at follow up and adjudicated per protocol [27].

**Fig. 1:**
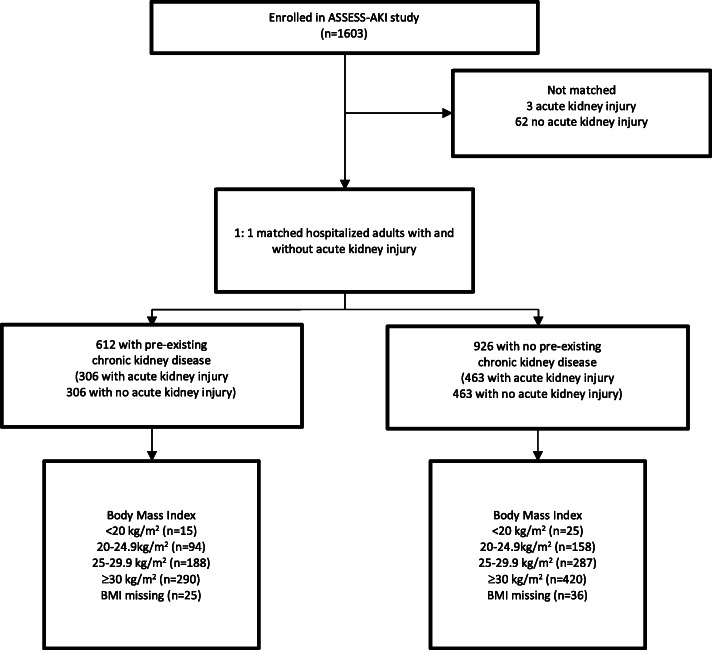
Formation of matched parallel cohort of adults surviving a hospitalization with and without acute kidney injury, including body mass index status at initial study visit

Statistical Analysis: Baseline co-variate distributions were tabulated with respect to baseline BMI categories and baseline AKI status. In the matched cohort, 61 participants did not have measurements of height and weight recorded at the baseline study visit (Fig. 1). Forty-two of these participants had height and weight measured at a subsequent study visit and this BMI was used. For the remaining 19 participants who did not have BMI recorded at any study visit, multiple imputation using chained equations was used. A total of 10 datasets were created, and imputation was informed by age, race, gender, and AKI diagnosis [32, 33]. Analyses were conducted using STATA version 14 (College Station, TX); *p* values were two-tailed and accepted type 1 error was 5% (α = 0.05).

Participants were considered at risk from the date of study entry until the to the first occurrence of the event of interest, death, or the end of available follow-up. Unadjusted incidence rates and 95% confidence intervals (CI) of incident CKD, CKD progression, MACE and ESRD, and death, per 1000 person years, were calculated for each combination of BMI and AKI groups. Kaplan-Meier curves were used to check for proportional hazards assumptions for each variable. Plots of Kaplan-Meier curves were additionally stratified by quantiles of log relative hazard to demonstrate that proportional hazards is a reasonable assumption. Time to event analyses using Cox proportional hazards models were conducted to estimate the relative hazard of events for the AKI group compared to the non-AKI group. Nested models were established to estimate adjusted associations based on a priori selection using matching criteria and other variables previously reported to be associated with kidney disease incidence, progression or cardiovascular events. Participants in the matched cohort remained unlinked and the matching factors age, pre-admission CKD status and CKD stage, diabetes, CVD, ICU admission, and urine albumin-to-creatinine ratio (uACR) were added in the first nested model (Model 1). The covariate uACR was logarithmically transformed for inclusion in the models. Further adjustments for race, gender, baseline eGFR as a continuous variable, sepsis, chronic heart failure (CHF) and smoking were included in the fully adjusted model (Model 2). For both models, baseline hazards were stratified by study center. Statistical interaction between AKI and BMI was modeled by including a product term for AKI and baseline BMI categories in the proportional hazards models. Models with, and without, the product term were compared using the Wald test. BMI subgroup-specific hazard ratios were obtained via linear combination of regression coefficients for main effect and BMI*AKI cross-product terms. Sensitivity analyses accounting for the competing risk of death were conducted using the Fine-Gray subdistribution hazard models [34], adjusted in a stepwise fashion, as in the primary analysis. Cumulative incidence plots using the Aalen-Johansen estimator and Fine-Gray models were created to compare the two methods, and to show the fraction of subjects in CKD and death states [35].

BMI was also modeled as a restricted cubic spline, generated from baseline BMI as a continuous variable, with knots at the 10th, 50th, and 90th percentiles. We constructed partial effect plots of the difference in hazard ratios between AKI and no AKI, which display the predicted outcome as a function of a single covariate while holding all other covariates constant for different levels of BMI. The plots are based on a multivariable Cox model that includes terms for AKI status, BMI as a restricted cubic spline, study center, baseline CKD status, ICU status, diabetes, age, cardiovascular disease, race, gender, smoking, sepsis, chronic heart failure, and the interaction between AKI status and BMI as a restricted cubic spline.

Secondary outcomes included death, MACE, ESRD, incident CKD, and CKD progression. Supplementary analyses included examining the effect of baseline BMI on the change in kidney function by calculating the log transformed slope of eGFR from baseline at each study visit, in participants with ≥2 eGFR values. Linear regression was used to evaluate the relationship between slope of eGFR and AKI status, modified by BMI. The reference group was normal BMI (20–24.9 kg/m^2^). Regression models included terms AKI status, BMI, baseline CKD, age, diabetes, CVD, race, gender, CHF, smoking, sepsis, ICU, uACR, the interaction term between AKI status and BMI, and were stratified by study center. Lastly, in order to examine the association between baseline BMI and CKD outcomes, multivariable time to event Cox models were constructed separately for the AKI and no-AKI groups with normal BMI as the reference group.

## Results

### Participant characteristics

There were 1603 participants in the ASSESS-AKI study cohort, including 772 who had AKI during hospitalisation and 831 who did not (Fig. 1) [36]. The study cohort included 1538 participants, matched 1-to-1 for AKI status, study center, baseline CKD status, ICU admission and co-morbidities. Three hundred-six matched pairs had pre-existing CKD, and 463 pairs did not. Table 1 displays the cohort characteristics, by AKI and BMI groups. The majority of participants were admitted to ICU during their index hospital admission and were former or are current smokers. Participants with AKI were more likely than those without AKI to have pre-existing CHF and sepsis during the index hospitalization.

**Table 1:** Baseline characteristics of adults with and without acute kidney injury, stratified by body mass index at study entry. Data presented as *n* (%) or mean (SD)

	No Acute Kidney Injury (*n* = 769)				Acute Kidney Injury (*n* = 769)			
	BMI < 20	BMI 20–24.9	BMI 25–29.9	BMI 30+	BMI < 20	BMI 20–24.9	BMI 25–29.9	BMI 30+
*n* (%)	21 (2%)	125 (17%)	269 (35%)	354 (46%)	16 (2%)	134 (17%)	223 (30%)	396 (51%)
Age	65.9 (13.5)	65.5 (13.9)	68.4 (12.2)	63.1 (11.9)	61.7 (15.1)	64.3 (16.0)	65.9 (12.7)	62.3 (11.2)
Women	14 (67%)	59 (47%)	84 (31%)	167 (47%)	7 (44%)	46 (34%)	49 (22%)	148 (37%)
Race								
White	19 (90%)	102 (82%)	241 (90%)	291 (82%)	11 (69%)	108 (81%)	181 (81%)	307 (77%)
Black	2 (10%)	9 (7%)	17 (6%)	50 (14%)	4 (25%)	16 (12%)	27 (12%)	70 (18%)
Other	0	14 (11%)	11 (4%)	13 (4%)	1 (6%)	10 (7%)	15 (7%)	19 (5%)
BMI (kg/m^2^)	18.4 (1.1)	23.0 (1.4)	27.5 (1.4)	36.3 (6.0)	18.4 (1.1)	22.8 (1.4)	27.3 (1.5)	37.5 (7.3)
eGFR ml/min/1.73m^2^								
Pre- admission								
	72 (23)	71 (24)	66 (22)	71 (26)	74 (29)	72 (29)	66 (25)	66 (25)
3-month baseline								
	77 (25)	73 (25)	70 (24)	74 (24)	74 (36)	70 (31)	64 (25)	65 (26)
Diabetes	3 (5%)	23 (18%)	84 (31%)	161 (45%)	4 (25%)	43 (32%)	87 (39%)	253 (64%)
CVD	7 (33%)	46 (37%)	127 (47%)	141 (40%)	5 (31%)	61 (46%)	103 (46%)	203 (51%)
CHF	2 (10%)	21 (17%)	46 (17%)	53 (15%)	6 (38%)	29 (22%)	55 (25%)	115 (29%)
Sepsis	0	4 (3%)	9 (3%)	13 (4%)	2 (13%)	27 (20%)	29 (13%)	60 (15%)
ICU	12 (57%)	80 (64%)	176 (65%)	205 (58%)	11 (69%)	104 (78%)	169 (76%)	261 (66%)
Smoker								
never	7 (33%)	47 (38%)	123 (46%)	149 (42%)	4 (25%)	53 (40%)	86 (39%)	165 (41%)
former	9 (43%)	53 (42%)	117 (43%)	166 (47%)	7 (44%)	50 (37%)	105 (47%)	182 (46%)
current	5 (2%)	23 (18%)	27 (10%)	35 (10%)	5 (31%)	31 (23%)	29 (13%)	47 (12%)
unknown	0	2 (2%)	2 (1%)	4 (1%)	0	0	3 (1%)	2 (1%)
AKI stage 1					15 (94%)	100 (75%)	174 (78%)	264 (67%)
AKI stage 2					0	18 (13%)	25 (11%)	75 (19%)
AKI stage 3					1 (6%)	16 (12%)	24 (11%)	57 (14%)

*AKI* acute kidney injury, *BMI* body mass index, *CHF* chronic heart failure, *CVD* cardiovascular disease, *eGFR* estimated glomerular filtration rate, *ICU* treated in intensive care unit

The distribution across BMI categories was similar for the AKI and no-AKI groups. 49% of the cohort had a BMI of 30 kg/m^2^ or greater. At baseline, the mean (SD) BMI was 31.6 (8.3) kg/m^2^ in the AKI group and 30.6 (7.0) kg/m^2^ in the non-AKI group (Table 1). Those with a BMI > 30 kg/m^2^ were more likely to experience more severe AKI, and had a higher incidence of diabetes, with similar age, race and gender distribution as other BMI categories. Pre-admission and study baseline eGFRs were similar, and did not differ widely as BMI increased.

### Outcomes

The unadjusted incidence rates for the combined CKD outcome, incident CKD, CKD progression, ESRD, death, and MACE are presented in Table 2. Unadjusted rates of the combined CKD outcome were lowest in those with normal weight and no AKI (17.7 per 1000 person years) and highest in the group with obesity and AKI (84.7 per 1000 person years). Mortality was higher in those with AKI compared to no AKI, with the highest mortality in the underweight AKI group (196.5 per 1000 person years), followed by the normal weight AKI group (64.9 per 1000 person years), AKI and overweight (60.8 per 1000 person years) and AKI and obesity (56.9 per 1000 person years). Unadjusted event rates for MACE were similar with and without AKI and across BMI groups.

**Table 2:** Incidence Rates per 1000 person years (95% confidence intervals) for outcomes during up to 8 years follow up in adults with or without acute kidney injury (AKI), stratified by body mass index

	BMI category			
	< 20 kg/m^2^	20–24.9 kg/m^2^	25–29.9 kg/m^2^	≥ 30 kg/m^2^
**Incident CKD (*n*** **= 201 events)**				
No AKI				
Events/total n	2/15	7/75	28/164	29/209
Rate	31.5 (7.9 to 125.8)	20.0 (9.5 to 42.0)	35.7 (24.7 to 51.7)	29.7 (20.6 to 42.7)
AKI				
Events	2/11	23/85	30/134	80/233
Rate	57.6 (14.4 to 230)	71.4 (47.5 to 108)	56.2 (39.3 to 80.3)	94.4 (75.8 to 118)
**CKD Progression (*n*** **= 64 events)**				
No AKI				
Events	1/6	1/50	7/105	11/145
Rate	44.9 (6.2 to 320)	4.6 (0.6 to 32.6)	14.8 (7.1 to 31.1)	18.4 (10.2 to 33.2)
AKI				
Events	0/5	9/49	11/89	24/163
Rate	0	47.1 (24.5 to 90.5)	32.7 (18.1 to 59.1)	38.7 (25.9 to 57.7)
**ESRD (*n*** **= 58 events)**				
No AKI				
Events	2/21	2/125	2/269	6/354
Rate	24.0 (6.0 to 96.1)	3.5 (0.9 to 13.8)	1.5 (0.4 to 8.1)	3.6 (1.6 to 8.1)
AKI				
Events	0/16	7/134	13/223	26/396
Rate	0	12.4 (5.9 to 26.1)	13.7 (8.0 to 23.6)	15.5 (10.5 to 22.7)
**Combined CKD event (*n*** **= 300 events)**				
No AKI				
Events	4/21	10/125	36/269	43/354
Rate	49.0 (18.4 to 130)	17.7 (9.5 to 33.0)	28.7 (20.7 to 39.7)	27.4 (20.3 to 36.9)
AKI				
Events	2/16	36/134	48/223	121/396
Rate	39.0 (9.7 to 155.9)	72.6 (52.3 to 101)	56.4 (42.5 to 74.8)	84.7 (70.9 to 101)
**Major Atherosclerotic Cardiovascular Events (MACE** ***n*** **= 129 events)**				
No AKI				
Events	2/21	13/125	26/269	28/354
Rate	24.1 (6.0 to 96.4)	24.3 (14.1 to 41.9)	22.0 (15.0 to 32.3)	18.5 (12.8 to 26.8)
AKI				
Events	2/21	8/134	25/223	25/396
Rate	37.8 (9.5 to 151.2)	15.2 (7.6 to 30.5)	28.1 (19.0 to 41.6)	15.4 (10.4 to 22.9)
**Death (*n*** **= 320 events)**				
No AKI				
Events	5/21	18/125	39/269	50/354
Rate	56.0 (23.3 to 135)	30.9 (19.5 to 49.0)	29.7 (21.7 to 40.6)	30.0 (22.8 to 39.6)
AKI				
Events	11/16	38/134	60/223	99/396
Rate	196.5 (109 to 355)	64.9 (47.2 to 89.2)	60.8 (47.2 to 78.4)	56.9 (46.7 to 69.3)

### Combined CKD outcome

**Fig. 2:**
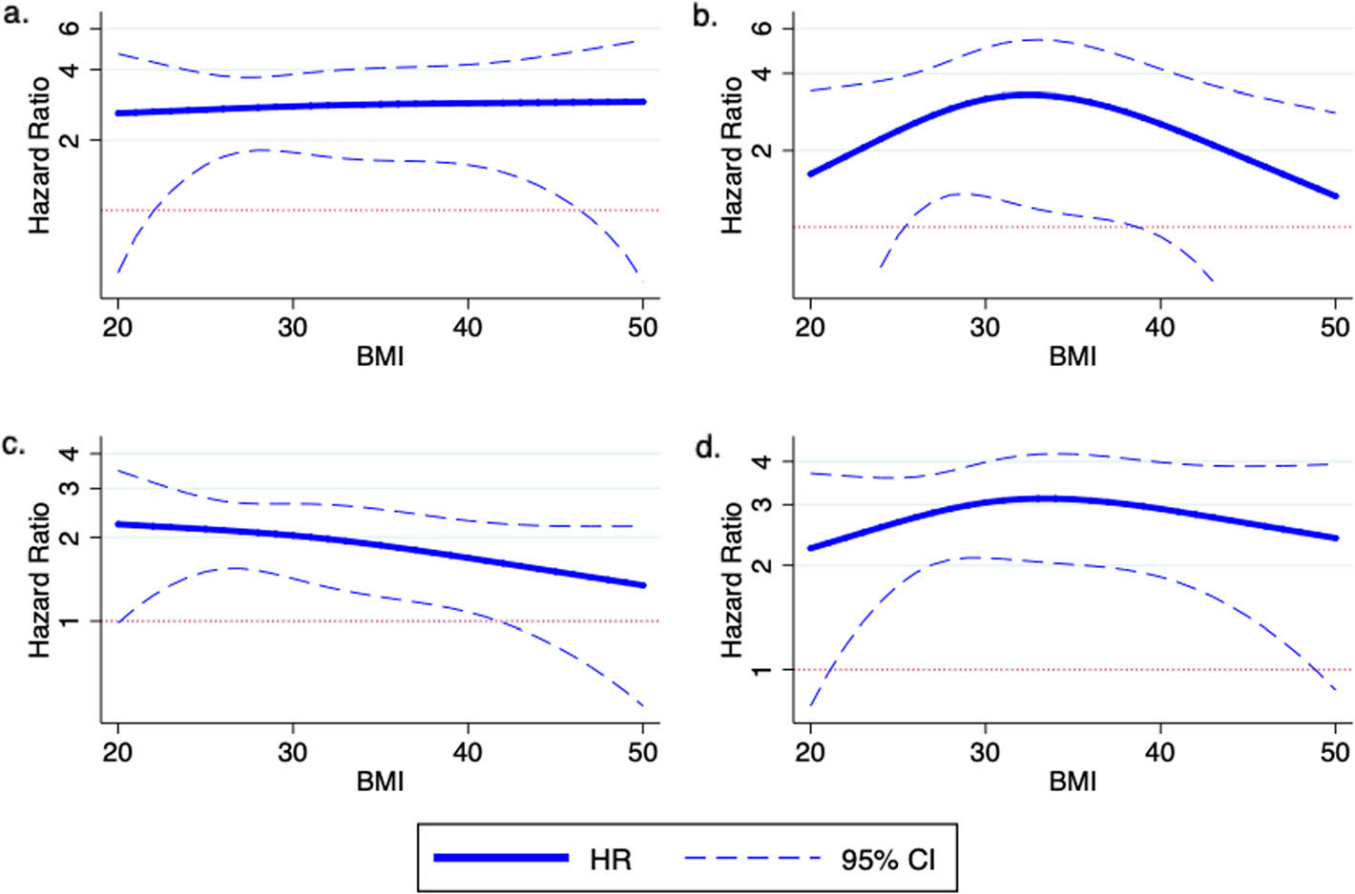
Partial effects plots of the difference in hazard ratio between AKI and no AKI across levels of BMI with **a** CKD incidence **b** CKD progression and ESRD **c** death and **d** combined CKD outcome. The plot is based on the multivariable Cox model that includes terms for AKI status, continuous BMI as a restricted cubic spline, study center, baseline CKD status, ICU status, diabetes, age, cardiovascular disease, race, gender, smoking, sepsis, chronic heart failure and the interaction between AKI status and BMI as a restricted cubic spline. Covariate values for the reference patient include age (65 years), race (white), gender (male), diabetes (no), hypertension (yes), smoker (former), CHF (no), CVD (no), ICU (no), and sepsis (no)

Median follow up time was 4.3 years (range 0.1–7.8 years) and there were 300 events, including 93 in the non-AKI group and 207 in the AKI group. In multivariable time to event analyses, AKI was associated with a higher risk for the combined CKD outcome, after adjustment for age, kidney disease, diabetes, CVD and ICU admission, and also in the fully adjusted model (Model 2 aHR 2.43; 95% CI 1.87 to 3.16). Compared to the non-AKI group, AKI was associated with an increased risk of the combined CKD outcome, however, we found no detectable modifying effect of obesity on the risk for the combined CKD outcome (Model 2, p for interaction = 0.3) (Table 3). The results were similar when BMI was included in the model as a continuous variable (Model 2 aHR 3.13 (95% CI 0.61 to 15.97, p for interaction = 0.7) and when BMI was modelled as a cubic spline term (Model 2 aHR 2.84 (95% CI 0.44 to 18.23), p for interaction = 0.6) (Fig. 2).

**Table 3:** Body mass index stratum specific time to event Hazard Ratios (HR) for the combined CKD event of CKD incidence, progression or End Stage Renal Disease in AKI group compared to non-AKI group including with interaction for body mass index

	Model 1 includes matching factors - age, CKD, diabetes, CVD, ICU, and urine albumin-to-creatinine ratio.		Model 2 Model 1 + race, sex, baseline eGFR, CHF, sepsis, and smoking	
Events (*n*)	300/1535		298/1521	
**Overall**	**HR (95% CI)**	***p***	**HR (95% CI)**	***p***
No AKI	1.0 (ref)	–	1.0 (ref)	–
AKI	2.73 (2.12, 3.51)	< 0.001	2.43 (1.87, 3.16)	< 0.001
**BMI**	**HR (95% CI)**	***p***	**HR (95% CI)**	***p***
≤ 20 kg/m^2^	0.80 (0.14, 4.38)	0.8	1.06 (0.19, 5.95)	0.9
20–24.9 kg/m^2^	3.90 (1.93, 7.89)	< 0.001	4.04 (1.99, 8.22)	< 0.001
25–29.9 kg/m^2^	2.12 (1.37, 3.28)	0.001	1.96 (1.26, 3.05)	0.003
≥ 30 kg/m^2^	3.06 (2.15, 4.37)	< 0.001	2.51 (1.74, 3.61)	< 0.001
p for interaction		0.2		0.3

*AKI *acute kidney injury, * BMI* body mass index, *CHF* chronic heart failure, *CI* confidence interval, *CKD* chronic kidney disease, *CVD* cardiovascular disease, *eGFR* estimated glomerular filtration rate, *ICU* admission to intensive care unit

Note: urine albumin-to-creatinine ratio is log transformed; both models stratify by study center

There were 320 deaths during follow up, including 112 in the non-AKI group and 208 in the AKI group, and median time to death was 4.9 years (range 0.1 to 7.8 years). In the sensitivity analysis including the competing risk of death, the subdistribution hazard risk of the combined CKD outcome in the fully adjusted model (Model 2) was SHR 2.27 (95% CI 1.76 to 2.92). The comparative cumulative incidence plots of the combined CKD outcomes using both Fine-Gray and Aalen-Johansen methods, are displayed in Fig. S1. Cumulative CKD and death events for the entire cohort (Fig. S2) and by AKI status (Fig. S3) illustrate the increased likelihood of CKD and death after AKI, compared to no AKI.

There was no detectable modifying effect of obesity on the association between AKI and the combined CKD outcome, after the adjustment for the competing risk of death, as the stratum specific risks with overweight and obesity sit within the 95% CI for the normal weight group (Table S1).

### Secondary outcomes

#### Incident CKD and CKD progression

The secondary analysis for the development of incident CKD was limited to the participants without CKD at baseline in the matched cohort (*n* = 924). There was an increased likelihood for developing incident CKD after AKI in the fully adjusted model (Model 2 aHR 2.63, 95% CI 1.91 to 3.61, *p* < 0.001), and the modifying effect of BMI was not detected (Model 2, *p* for interaction = 0.38). For CKD progression, the analysis was limited to those with CKD at baseline. In adjusted models, the risk for CKD progression after AKI was marginally increased (Model 2 aHR 1.62 (95% CI 0.90 to 2.92), and again, we were unable to detect a modifying effect by BMI (Model 2, *p* for interaction = 0.14).

#### End stage renal failure, cardiovascular events and death

In fully adjusted models, AKI was associated with an increased risk for death (Model 2 aHR 1.84, 95% CI 1.44 to 2.36), and ESRD (Model 2 aHR 2.29, 95% CI 1.12 to 4.66), but not MACE (Model 2 aHR 0.57, 95% CI 0.23 to 1.42). There was no detectable modifying effect of obesity on the relationship between AKI and risk of death (*p* for interaction = 0.5) or ESRD (*p* for interaction = 0.08) in the fully adjusted models. BMI stratum specific HRs for CKD incidence, CKD progression, ESRD and death are listed in Table S2.

#### Rate of change in eGFR

There was no association between AKI and the rate of change in eGFR for the combined CKD event in models including an interaction term for BMI, either as a categorical variable or as a continuous variable (Model 2 β = − 0.03, 95% CI − 0.12 to 0.06; *p* for interaction = 0.91) (Table S3).

#### BMI and risk for CKD in study cohort

The association between BMI and subsequent risk of the combined CKD outcome was also examined in this cohort of hospitalized patients, with or without an AKI event. In models adjusted for age, race and gender, there was no evidence for an increased risk for the combined CKD outcome, with higher BMI, in the non-AKI or AKI groups (Table S4). However, there was a trend towards an increased risk for the combined CKD event in the low BMI non-AKI group (aHR 2.7, 95% CI 0.9 to 8.8).

## Discussion

In this prospective matched cohort study, the fully adjusted risk of developing the combined CKD outcome was almost 2.5 times higher in those who experienced AKI, compared to those who did not. This was largely driven by increased CKD incidence after AKI. The risk for CKD outcomes was higher in the present study than in generally healthy, post AKI, community-based cohorts, indicating that the higher overall risk potentially blunted the hypothesized modifying effect of BMI. The majority of participants in our study were admitted to the ICU during hospitalization, indicating a high severity of illness and likely acute inflammation, which may have a greater impact on longer term outcomes than the more modest effect of high BMI. Risk for MACE was not different with or without AKI in this cohort, indicating that an acute insult to the kidneys did not impact upon the risk for cardiovascular events after hospitalization in this population.

We were unable to detect a modifying effect of BMI on the association of AKI and the combined outcome of incident CKD, CKD progression or ESRD in this cohort, even after adjustment for the competing risk of death. In separate models examining incident CKD, CKD progression and mortality, there was also no detectable modifying effect of BMI on the primary study finding that AKI is independently associated with a higher risk of the development of CKD, CKD progression, and mortality with up to 8 y of follow-up. Several studies have examined the effect of BMI on mortality after AKI over the short term, although these were retrospective [18, 19, 37] or prospective with no comparator group [17, 38]. Some studies show that up to 6 months post AKI, higher BMI is protective against mortality, compared to lower BMI [17–19, 38]. Our findings concur with the largest study prospective study, which found that high body mass index (BMI) did not modify the risk of mortality 1-year post discharge in a cohort of patients with AKI admitted to critical care, compared to those without AKI [37].

This study explicitly examined how body mass index may modify the risk of further kidney damage after an episode of AKI. In a small feasibility study, obesity was not associated with the relative risk of CKD development or progression 12 months after AKI [39]. The present study extends the follow up time beyond 12 months, and adds to the literature on the role of BMI on CKD outcomes after AKI. In this study we were unable to detect an effect to suggest that mechanisms of progressive kidney damage after AKI are adversely modified by larger body size in this AKI survival cohort. Given the wide confidence intervals for the associations examined, we cannot discount that our study may be underpowered to detect an interaction with BMI in this cohort. BMI distribution was heavily skewed towards obesity, with over 50% of the cohort in the BMI 30+ category, indicating a possible survival advantage after AKI with higher BMI. Potentially, higher BMI may lower the risk for CKD outcomes, as the stratum specific hazard ratios display a consistent trend for lower risk with higher BMI. Whilst we were unable to detect evidence for a possible deleterious or protective effect, a sufficiently powered study may support findings contrary to the original hypothesis. Other, unaccounted for variables, such as statin use, and antihypertensive medications, may be more likely to be prescribed in higher BMI patients, and may contribute to a potential protective effect of higher BMI on CKD outcomes after AKI, due to a higher level of medical management.

Recent evidence which added genetic information to risk models, demonstrates that the independent risk of obesity on CKD outcomes is likely causal, however the associated risk is lower than observational studies using conventional methods suggested [40]. Furthermore, much of the BMI associated risk for CKD in the genetic models is attributable to diabetes and hypertension [40]. In longitudinal cohort models such as ours, which included diabetes and hypertension as potential confounders, plus the added insult of AKI, it is possible that the small impact attributable to BMI is made negligible by the AKI, and possibly also by the episode of illness leading to hospitalization, as we also did not find evidence of BMI as a risk factor for CKD in this population, with or without AKI. In this same cohort, proteinuria has been identified to increase the risk of kidney disease progression [41]. Weight loss interventions in those with obesity and CKD have been demonstrated to reduce proteinuria [42], so there may still be a role for studying obesity interventions after AKI in those with proteinuria, to establish if reducing proteinuria alters CKD risk.

The strengths of this study include the inclusion criteria of pre-admission kidney function, the prospective matched cohort design, which matches on potential confounders, and the systematic follow up which included standardised measurement of height and weight at the baseline study visit, and measurement of kidney function at regular intervals. The statistical model was designed a-priori with established clinical factors included as potential confounders. However, there remain factors that may have limited the analyses, such as the very small underweight group with a larger number of events, the wide range of BMI which may have increased the variance in the sample and reduced the power, and the use of BMI as a proxy for obesity. No other measure of body composition or obesity, such as waist circumference, waist to hip ratio or fat mass derived from body composition analysis was available.

BMI was a calculated from a single measure of weight and height at the baseline study visit 3 months post AKI. Weight loss during acute illness is common, and BMI at the first study visit may not have reflected usual BMI. The difference between usual BMI and the BMI at baseline is unknown and is a limitation in the study, as acute illness associated weight loss may have resulted in categorization to a lower BMI category than usual BMI would have. Whilst the matched cohort design is a strength in a longitudinal cohort study, the matching process may not always proceed as planned. Our cohort was primarily matched for study center and baseline CKD status (eGFR > or < 60 ml/min/ 1.73m^2^), and although participants with AKI were attempted to be matched with non-AKI participants for age, cardiovascular disease, diabetes, category of baseline eGFR, and ICU admission, matching on these factors was not always possible, and our AKI group had a higher prevalence of diabetes, ICU admission, CKD and also more men and more participants with Black race, than the no AKI group. Further adjustment was included in the models to account for the independent effects of these factors on CKD outcomes.

In summary, we found no detectable modifying effect of higher BMI on the relationship between AKI and future risk of CKD outcomes, and we did not find evidence to suggest an association between obesity and the development or progression of CKD in this cohort followed from an index hospitalization after acute illness. Future studies addressing established risk factors such as proteinuria, with and without weight loss as a treatment component, may be useful in determining the role of obesity among AKI survivors.

## Supplementary Information

**Additional file 1: Table S1**: Body mass index stratum specific Subdistribution Hazard Ratios (SHR) for the combined CKD event of CKD incidence, progression or End Stage Renal Disease in AKI group compared to non-AKI group, including the competing risk of death. **Table S2**: Body Mass Index stratum specific time to event adjusted hazard ratios (HR) for outcomes with AKI compared to no AKI in a matched cohort of hospitalized participants. **Table S3**: Body Mass Index stratum specific association between change in eGFR during follow up and the combined CKD event in a matched cohort of hospitalized participants. **Table S4**: Time to event Hazard Ratios (HR) for the combined CKD event of CKD incidence, progression or end stage renal disease by body mass index. **Figure S1**. Cumulative incidences for CKD comparing the Aalen-Johansen method and Fine-Gray models, stratified by AKI status at baseline. **Figure S2**. Aalen-Johansen cumulative incidence for CKD and death in all subjects. **Figure S3**. Aalen-Johansen cumulative incidence for CKD and death, stratified by AKI status at baseline.

## Data Availability

The data that support the findings of this study are available from the ASSESS-AKI Ancillary Study committee, but restrictions apply to the availability of these data, which were used with permission for the current study, and so are not publicly available. Data are however available from the authors Dr. Helen MacLaughlin, and Dr. Cassianne Robinson-Cohen, upon reasonable request and with permission of the ASSESS AKI Study Committee.
