# Supplementary material for: Body mass index and chronic kidney disease outcomes after acute kidney injury: a prospective matched cohort study
Source: BMC Nephrol. 2021 May 28;22:200. doi: 10.1186/s12882-021-02400-3 (PMC8161937; doi:10.1186/s12882-021-02400-3)
Supplement: Supplementary file 1 — Additional file 1: Table S1: Body mass index stratum specific Subdistribution Hazard Ratios (SHR) for the combined CKD event of CKD incidence, progression or End Stage Renal Disease in AKI group compared to non-AKI group, including the competing risk of death. Table S2: Body Mass Index stratum specific time to event adjusted hazard ratios (HR) for outcomes with AKI compared to no AKI in a matched cohort of hospitalized participants. Table S3: Body Mass Index stratum specific association between change in eGFR during follow up and the combined CKD event in a matched cohort of hospitalized participants. Table S4: Time to event Hazard Ratios (HR) for the combined CKD event of CKD incidence, progression or end stage renal disease by body mass index. Figure S1. Cumulative incidences for CKD comparing the Aalen-Johansen method and Fine-Gray models, stratified by AKI status at baseline. Figure S2. Aalen-Johansen cumulative incidence for CKD and death in all subjects. Figure S3. Aalen-Johansen cumulative incidence for CKD and death, stratified by AKI status at baseline. [file 12882_2021_2400_MOESM1_ESM.docx]

Supplementary Material

**Body mass index and chronic kidney disease outcomes after acute kidney injury:**

**a prospective matched cohort study**

Helen L MacLaughlin, RD, PhD^1,2^, Mindy Pike, MPH^3^, Nicholas M Selby, MD^4^, Edward Siew, MD, MSci^3^, Vernon M Chinchilli PhD^5^, Andrew Guide, MS^6^, Thomas G Stewart, PhD^6^, Jonathan Himmelfarb, MD^7^, Alan S Go MD^8,9^, Chirag R Parikh MBBS PhD^10^, Nasrollah Ghahramani MD^11^, James Kaufman, MD^12^, T Alp Ikizler, MD MS^3^, Cassianne Robinson-Cohen, PhD^3^ for the ASSESS-AKI Study Investigators

1. Queensland University of Technology, School of Exercise and Nutrition Sciences, Brisbane, Australia h.maclaughlin@qut.edu.au
2. Royal Brisbane and Women’s Hospital, Herston, Qld, Australia
3. Division of Nephrology, Vanderbilt University Medical Center, Nashville, TN, USA
4. Centre for Kidney Research and Innovation, University of Nottingham, Derby, UK
5. Division of Biostatistics and Informatics, Pennsylvania State University, Hershey, PA, USA
6. Department of Biostatistics, Vanderbilt University School of Medicine, Nashville, TN, USA
7. Division of Nephrology, University of Washington, Seattle, WA, USA
8. Kaiser Permanente Northern California, Oakland, CA, USA
9. University of California, San Francisco, San Francisco, CA, USA
10. Division of Nephrology, Johns Hopkins School of Medicine, Baltimore, Maryland
11. Division of Nephrology, Department of Medicine, Penn State College of Medicine, Hershey, PA, USA
12. Renal Section, Veterans Affairs New York Harbor Health Care System and New York University School of Medicine, New York, NY, USA

Table S1: Body mass index stratum specific Subdistribution Hazard Ratios (SHR) for the combined CKD event of CKD incidence, progression or End Stage Renal Disease in AKI group compared to non-AKI group, including the competing risk of death

|  | Model 1  matching factors - age, CKD, diabetes, CVD, ICU, and uACR | | Model 2  Model 1 + race, sex, baseline GFR, CHF, sepsis, and smoking | |
| --- | --- | --- | --- | --- |
| Events (n) | 230/1538 | | 228/1521 | |
| Competing events (n) | 320/1538 | | 317/1521 | |
| **Overall** | **SHR (95% CI)** | **p** | **SHR (95% CI)** | **p** |
|  | 2.50 (1.96 to 3.18) | <0.001 | 2.27 (1.76 to 2.92) | <0.001 |
| **BMI** | **HR (95% CI)** | **p** | **HR (95% CI)** | **p** |
| ≤20 kg/m^2^ | 0.65 (0.12 to 3.16) | 0.6 | 0.81 (0.15, 4.46) | 0.8 |
| 20-24.9 kg/m^2^ | 3.54 (1.78, 7.03) | <0.001 | 3.72 (1.85, 7.49) | <0.001 |
| 25-29.9 kg/m^2^ | 1.92 (1.26, 2.94) | 0.003 | 1.78 (1.15, 2.76) | 0.009 |
| ≥30 kg/m^2^ | 2.84 (2.02, 3.98) | <0.001 | 2.40 (1.68, 3.41) | <0.001 |

BMI – body mass index, CHF – chronic heart failure; CI – confidence interval; CKD – chronic kidney disease; CVD – cardiovascular disease; eGFR – estimated glomerular filtration rate; ICU – admission to intensive care unit; uACR – urine albumin to creatinine ratio

Note: uACR is log transformed; all models are stratified by study center

Table S2: Body Mass Index stratum specific time to event adjusted hazard ratios (HR) for outcomes with AKI compared to no AKI in a matched cohort of hospitalized participants

|  | Model 1  Matching factors - age, baseline CKD, diabetes, CVD, and ICU and uACR | | Model 2  Model 1 + race, sex, baseline eGFR, CHF, sepsis, and smoking | | |
| --- | --- | --- | --- | --- | --- |
| **Incident CKD** (n = 924) | | |  | | |
| Events | 201 | | 200 | | |
| **BMI** | **HR (95% CI)** | **p** | **HR (95% CI)** | | **p** |
| ≤20 kg/m^2^ | 2.75 (0.38 to 19.77) | 0.3 | 3.04 (0.42 to 22.22) | | 0.3 |
| 20-25 kg/m^2^ | 3.83 (1.63 to 8.98) | 0.002 | 4.21 (1.76 to 10.05) | | 0.001 |
| 25-29.9 kg/m^2^ | 1.81 (1.08 to 3.05) | 0.03 | 1.82 (1.07 to 3.11) | | 0.03 |
| ≥30 kg/m^2^ | 3.32 (2.15 to 5.12) | <0.001 | 2.94 (1.87 to 4.61) | | <0.001 |
| p for interaction |  | 0.28 |  | | 0.38 |
| **CKD progression** (n = 612) | | |  | | |
| Events | 63 | | 62 | | |
| **BMI** | **HR (95% CI)** | **p** | **HR (95% CI)** | **p** | |
| ≤20 kg/m^2^ | - | - | - | - | |
| 20-25 kg/m^2^ | 6.60 (0.81 to 53.53) | 0.07 | 7.34 (0.87 to 62.15) | 0.07 | |
| 25-29.9 kg/m^2^ | 1.99 (0.74 to 5.36) | 0.17 | 1.45 (0.52 to 4.09) | 0.4 | |
| ≥30 kg/m^2^ | 1.39 (0.66 to 2.95) | 0.4 | 1.38 (0.64 to 2.98) | 0.4 | |
| p for interaction |  | 0.16 |  | 0.14 | |
| **ESRD** (n = 1538) | | |  | | |
| **Events** | 58 | | 58 | | |
| **BMI** | **HR (95% CI)** | **p** | **HR (95% CI)** | **p** | |
| ≤20 kg/m^2^ | - | - | - | - | |
| 20-25 kg/m^2^ | 2.27 (0.46 to 11.16) | 0.3 | 1.48 (0.28 to 7.86) | 0.6 | |
| 25-29.9 kg/m^2^ | 7.49 (1.65 to 34.10) | 0.009 | 4.53 (0.96 to 21.38) | 0.06 | |
| ≥30 kg/m^2^ | 2.80 (1.12 to 6.96) | 0.02 | 2.21 (0.82 to 5.91) | 0.1 | |
| p for interaction |  | 0.02 |  | 0.08 | |
| **Death (n = 1538)** | | |  | | |
| **Events** | 320 | | 317 | | |
| **BMI** | **HR (95% CI)** | **p** | **HR (95% CI)** | **p** | |
| ≤20 kg/m^2^ | 3.44 (1.19 to 9.96) | 0.02 | 3.00 (1.02 to 8.80) | 0.05 | |
| 20-24.9 kg/m^2^ | 2.04 (1.16 to 3.60) | 0.01 | 1.86 (1.05 to 3.29) | 0.03 | |
| 25-29.9 kg/m^2^ | 2.32 (1.54 to 3.50) | <0.001 | 2.17 (1.43 to 3.30) | <0.001 | |
| ≥30 kg/m^2^ | 1.85 (1.30 to 2.62) | 0.001 | 1.52 (1.06 to 2.18) | 0.02 | |
| p for interaction |  | 0.6 |  | 0.5 | |

BMI – body mass index, CHF – chronic heart failure; CI – confidence interval; CKD – chronic kidney disease; CVD – cardiovascular disease; eGFR – estimated glomerular filtration rate; ICU – admission to intensive care unit; uACR – urine albumin to creatinine ratio

Note: uACR is log transformed; all models are stratified by study center

| Table S3: Body Mass Index stratum specific association between change in eGFR during follow up and the combined CKD event in a matched cohort of hospitalized participants   \|  \| **Mean (SD)** \| \| \| **β Coefficient (95% CI)**  for AKI compared to no AKI \| \| \| --- \| --- \| --- \| --- \| --- \| --- \| \|  \| \| **No AKI** \| **AKI** \| Model 1  Matching factors center, age, baseline CKD, diabetes, CVD, and ICU \| Model 2  Model 1 + race, sex, baseline eGFR, CHF, sepsis, and smoking \| \| **BMI Category** \| \|  \|  \|  \|  \| \| ≤20 kg/m^2^ \| \| -0.05 (0.14) \| -0.14 (0.28) \| -0.08 (-0.22, 0.07) \| -0.07 (-0.22, 0.07) \| \| 20-24.9 kg/m^2^ \| \| -0.03 (0.09) \| -0.05 (0.16) \| -0.02 (-0.07, 0.03) \| -0.02 (-0.07, 0.04) \| \| 25-29.9 kg/m^2^ \| \| -0.03 (0.11) \| -0.08 (0.42) \| -0.05 (-0.09, -0.02) \| -0.06 (-0.09, -0.02) \| \| ≥30 kg/m^2^ \| \| -0.03 (0.10) \| -0.06 (0.18) \| -0.02 (-0.06, 0.01) \| -0.03 (-0.06, 0.00) \| \| p for interaction \| \|  \|  \| 0.56 \| 0.58 \| \|  \| \|  \|  \|  \|  \| \| Continuous BMI \| \| - \| - \| -0.04 (-0.13, 0.05) \| -0.03 (-0.12, 0.06) \| \| p for interaction \| \|  \|  \| 0.95 \| 0.91 \| \|  \| \|  \|  \|  \|  \| |
| --- | --- | --- | --- | --- | --- | --- | --- | --- | --- | --- | --- | --- | --- | --- | --- | --- | --- | --- | --- | --- | --- | --- | --- | --- | --- | --- | --- | --- | --- | --- | --- | --- | --- | --- | --- | --- | --- | --- | --- | --- | --- | --- | --- | --- | --- | --- | --- | --- | --- | --- | --- | --- | --- | --- | --- | --- | --- | --- | --- | --- | --- | --- | --- | --- | --- | --- | --- | --- | --- | --- | --- | --- |

Combined CKD event = CKD incidence, CKD progression or end stage renal disease, AKI – acute kidney injury, BMI – body mass index, CHD – chronic heart failure, CKD – chronic kidney disease, CVD – cardiovascular disease, eGFR – estimated glomerular filtration rate, ICU – intensive care unit, SD – standard deviation

Table S4: Time to event Hazard Ratios (HR) for the combined CKD event of CKD incidence, progression or end stage renal disease by body mass index

|  | **HR (95% CI)**  Adjusted for age, race and sex | **p** | **HR (95% CI**)  Adjusted for age, race and sex | **p** |
| --- | --- | --- | --- | --- |
| Events (n) | 230/1538 |  | 228/1521 |  |
| **BMI (**kg/m^2^) | **No AKI** |  | **AKI** |  |
| ≤20 | 2.7 (0.9 to 8.8) | 0.09 | 0.6 (0.2 to 1.83) | 0.1 |
| 20-24.9 | 1 |  | 1 |  |
| 25-29.9 | 1.5 (0.7 to 3.0) | 0.3 | 0.8 (0.4 to 1.6) | 0.2 |
| ≥30 | 1.2 (0.6 to 2.5) | 0.5 | 1.0 (0.5 to 2.0) | 0.6 |

BMI – body mass index; CI – confidence interval

Supplemental Figures

Figure S1. Cumulative incidences for CKD comparing the Aalen-Johansen method and Fine-Gray models, stratified by AKI status at baseline.

Figure S2. Aalen-Johansen cumulative incidence for CKD and death in all subjects.

Figure S3. Aalen-Johansen cumulative incidence for CKD and death, stratified by AKI status at baseline.


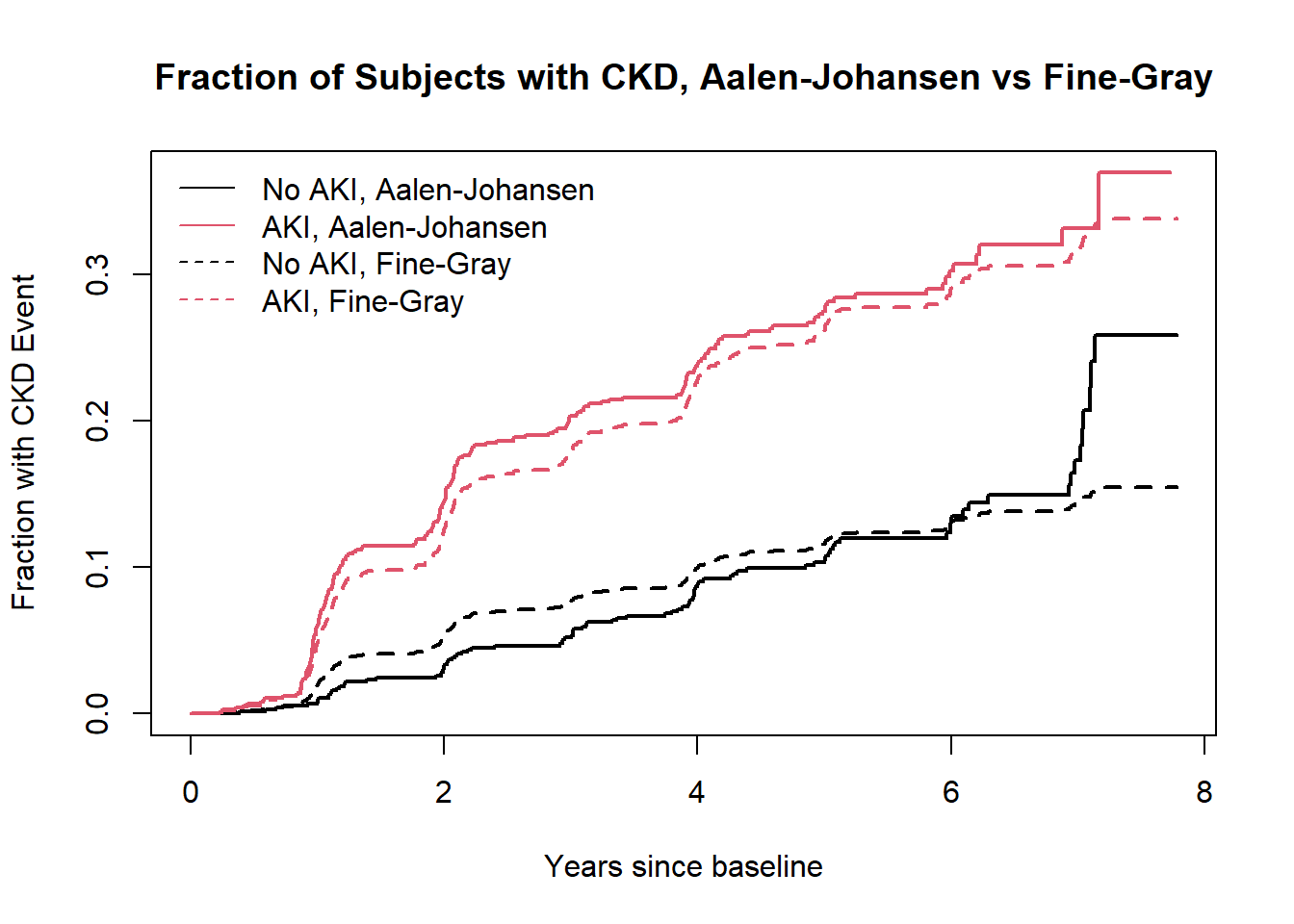


Figure S1.

**
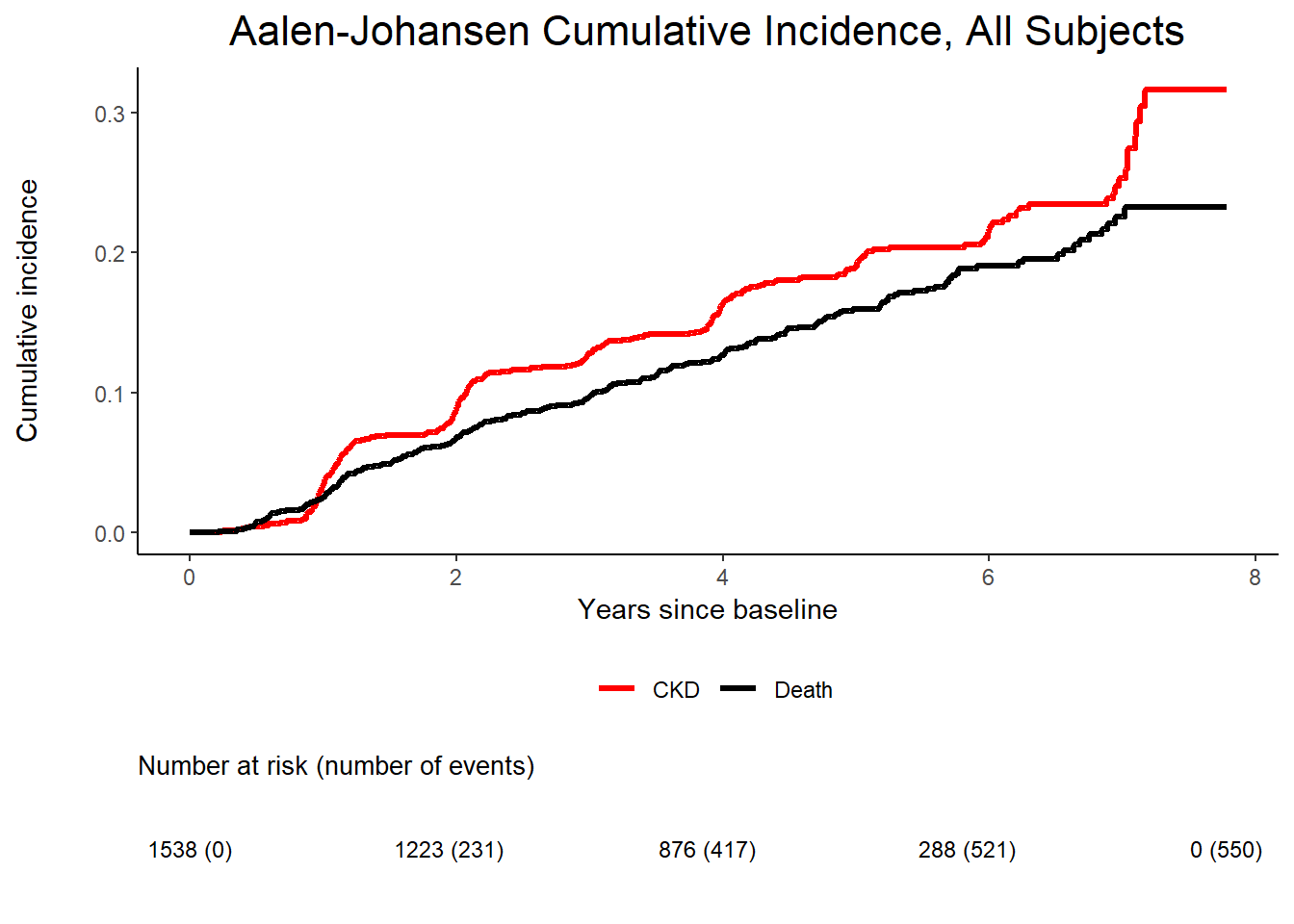
**

Figure S2.


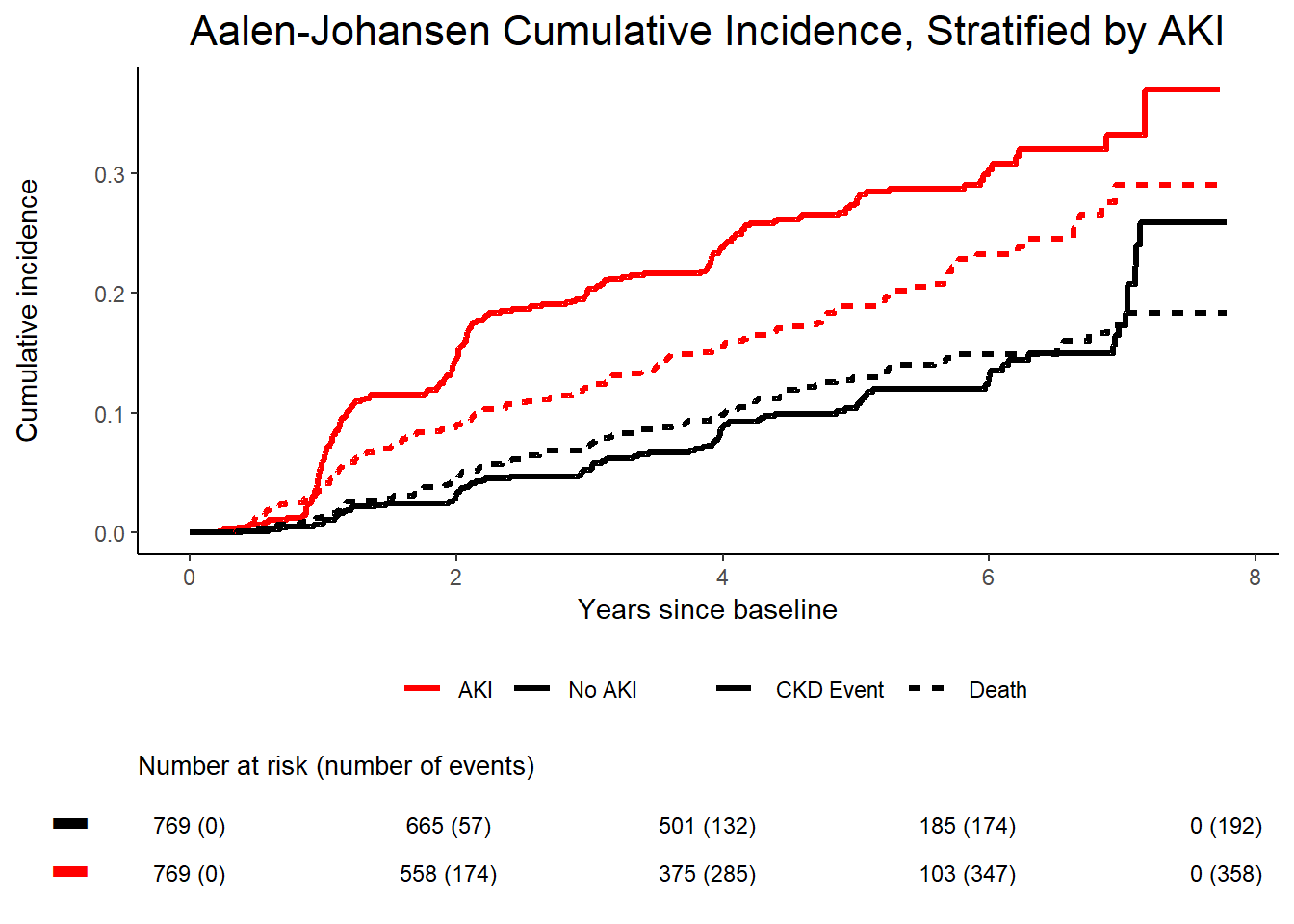


Figure S3.
